# Low attenuation areas in necrotizing soft tissue infection

**DOI:** 10.1002/ccr3.8566

**Published:** 2024-03-01

**Authors:** Masanori Kawataki, Yuta Oda

**Affiliations:** ^1^ Department of Respiratory Medicine, Ohara Healthcare Foundation Kurashiki Central Hospital Okayama Japan; ^2^ Department of Critical Care Medicine, Ohara Healthcare Foundation Kurashiki Central Hospital Okayama Japan

**Keywords:** abscess, low attenuation area, muscle necrosis, necrotizing soft tissue infection

## Abstract

Necrotizing Soft Tissue Infection can be challenging to differentiate from abscesses based on computed tomography imaging findings only, so it is crucial to perform surgical debridement as early as possible.

## 1 INTRODUCTION

Necrotizing Soft Tissue Infection (NSTI) is a severe infection in which necrosis spreads to the epidermis, subcutaneous tissue, and superficial fascia resulting in sepsis and a fatal situation.^1^ Mortality has been reported to increase with delayed surgical debridement,^1^ and it is essential to be suspect. Herein, we report a challenging case to differentiate from an abscess by imaging.

## 2 CASE REPORT

**FIGURE 1:**
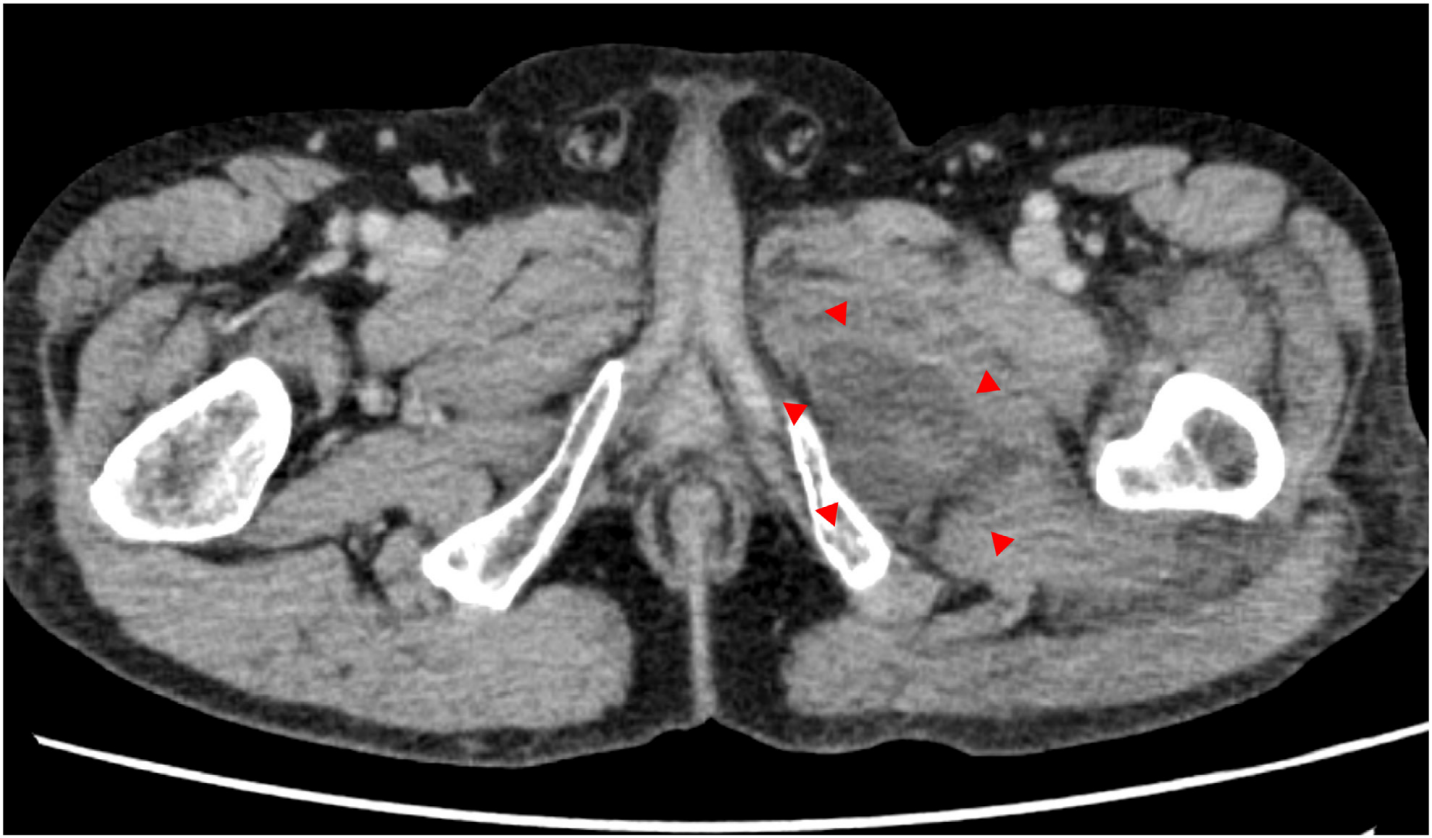
Contrast‐enhanced computed tomography revealed low attenuation in the left adductor muscle group (red arrows). No fluid retention or increased fat tissue density was observed.

A 72‐year‐old male presented to our hospital with left hip joint pain and general fatigue for 4 days. He had a medical history of erythroderma and was taking prednisolone 5 mg. Past medical history is hypertension, and he takes amlodipine. There was neither a recent drug initiation, surgical history, nor recent trauma history. The examination revealed pain in the left hip joint during passive movement but no erythema or blistering of the skin. No visible minor wounds on the extremities or trunk were present. Laboratory tests revealed C‐reactive protein of 30.96 mg/dL, white blood cell of 10.7 × 10^9^/L, Hb 14.9 g/dL, Na 141 mmol/L, creatinine 2.52 mg/dL, and glucose of 112 mg/dL. Contrast‐enhanced computed tomography (CT) showed low attenuation in the left adductor muscle group. No fluid retention or increased fat tissue density was observed (Figure 1). Intramuscular abscess was suspected, and surgical debridement was performed. Surgical debridement revealed cloudy exudate in the superficial fascia and synovial sac. The diagnosis of NSTI was subsequently confirmed. Vancomycin (initial dose 1000 mg q12h (≈15 mg/kg)), meropenem, and clindamycin were started. Because of hypotension and decreased renal function, we administered noradrenaline and temporarily continuous hemodialysis. Renal function improved, continuous hemodialysis was withdrawn, and creatinine improved to 0.7 mg/dL. Methicillin‐resistant Staphylococcus aureus (MRSA) was detected in joint fluid and tissue cultures. Meropenem and clindamycin were terminated after MRSA was detected. Antibiotic therapy was continued for approximately 12 weeks, and he was transferred to another hospital for rehabilitation.

## 3 DISCUSSION

NSTI on CT show typically gas along the fascia plane, fat stranding, increased density, edema and thickening of the fascia, obscure appearance of the fascial surface, non‐enhancement of fascia, and fluid retention. Meanwhile, CT findings of muscle necrosis show low attenuation and are associated with muscle edema.^2^

In the abscess, fluid attenuation on CT is a collection circumscribed by an enhanced, irregular, thin wall. Moreover, the surrounding tissue can develop edema and a low‐density area.^2^ Therefore, CT imaging alone may not be adequate for the diagnosis of NSTI, so distinguishing NSTI from abscess based on CT alone may be difficult, especially if the findings are atypical. However, physical examination findings may help distinguish between the two. It is not difficult to recall NSTI if erythema, blistering, skin necrosis, or fluctuance are present. In the absence of these findings, pain‐out‐of‐proportion has also been reported to be helpful in the diagnosis of NSTI.^3^ In this case, the patient had no erythema or blister formation but had very painful findings, and NSTI should have been suspected. Conducting a surgical consultation as early as possible is critical if the imaging findings are not typical.

## AUTHOR CONTRIBUTIONS

**Masanori Kawataki:** Writing – original draft. **Yuta Oda:** Writing – review and editing.

## FUNDING INFORMATION

The authors did not receive any grant support.

## CONFLICT OF INTEREST STATEMENT

None declared.

## ETHICS STATEMENT

Appropriate written informed consent was obtained from the patient for the publication of this case report and images. The consent form signed by the patient is held at our institution. The institutional review board approved this study.

## Data Availability

All data relevant to the study are included in the article.
